# Hesperetin Induces Ferroptosis-Like Response in *Saccharomyces cerevisiae*

**DOI:** 10.4014/jmb.2602.02027

**Published:** 2026-03-26

**Authors:** Huiwon Jang, Dong Gun Lee

**Affiliations:** 1 School of Life Sciences, BK21 FOUR KNU Creative BioResearch Group, Kyungpook National University, Daegu 41566, Republic of Korea; 2 Institute of Life Science and Biotechnology, Kyungpook National University, Daegu 41566, Republic of Korea

**Keywords:** Hesperetin, *Saccharomyces cerevisiae*, Hydroxyl radical, Lipid peroxidation, Iron-dependent, Ferroptosis-like response

## Abstract

Hesperetin has been reported to exhibit multiple beneficial activities, including anti-inflammatory and antimicrobial effects. It has also been shown to induce intracellular reactive oxygen species (ROS). However, its mode of action in fungi remains unclear. Therefore, this study aimed to clarify the underlying mechanisms of hesperetin using *Saccharomyces cerevisiae* as a model organism. Many antimicrobial compounds exert their effects by inducing oxidative damage in microbial cells. In this study, hesperetin increased intracellular reactive oxygen species in *S. cerevisiae*. This ROS accumulation was accompanied by glutathione depletion, indicating impaired antioxidant capacity and disrupted redox balance. Increased oxidative stress was also associated with an expanded pool of reactive iron, which can drive iron-dependent chemistry to generate highly reactive hydroxyl radicals. These radicals can initiate lipid peroxidation, leading to the accumulation of lipid hydroperoxides. Notably, these oxidative and lipid damage phenotypes were suppressed by ferrostatin-1, a ferroptosis inhibitor. In addition, apoptosis-associated hallmarks, including caspase activation and DNA fragmentation, were not observed under the same conditions. These findings indicate that hesperetin induces ferroptosis-like responses in *S. cerevisiae*, providing mechanistic insight into its antifungal effects.

## Introduction

Flavonoids are ubiquitous plant-derived secondary metabolites belonging to the polyphenol family. They are abundant in a wide range of plant-based foods, including fruits, vegetables, and herbs [1]. These compounds act as defensive molecules that help environmental challenges such as ultraviolet radiation, pathogen infection, and other environmental stresses, thereby contributing to diverse biological activities [2]. Among flavonoids, hesperetin is a representative flavanone [3]. It is predominantly found in citrus peels and related byproducts [4, 5]. It has been reported to exhibit multiple physiological activities [6]. These include antimicrobial and anti-inflammatory effects [6, 7]. It can also modulate cellular responses by influencing redox homeostasis and promoting the generation of oxidative stress [8]. Reactive oxygen species act as a key mediator of antimicrobial activity by inducing oxidative stress that can compromise essential cellular components [9]. Accordingly, this study investigates the antifungal activity of hesperetin against *Saccharomyces cerevisiae*.

ROS are oxidative molecules and radical species that are generated when molecular oxygen (O_2_) undergoes partial reduction instead of complete reduction to water [10]. Major ROS include superoxide (O_2_^-^•), hydrogen peroxide (H_2_O_2_), and the hydroxyl radical (•OH), which differ in their reactivity, stability, and cellular targets [11, 12]. The biological effects of ROS strongly depend on their concentration and duration [13]. At low levels, ROS act as signaling mediators that regulate physiological processes such as stress adaptation, metabolic control, and defense responses [14, 15]. However, when ROS production exceeds the capacity of antioxidant systems and ROS accumulate, they trigger oxidative stress and induce widespread cellular damage such as membrane damage and protein denaturation [16, 17]. Consequently, the cumulative and overlapping damage caused by excessive ROS can push cells beyond the point of recovery and ultimately induce cell death [18].

When downstream damage accumulates in response to reactive molecules such as ROS, cells may be driven beyond functional impairment toward a characteristic, regulated pattern of death [19]. This regulated process is referred to as programmed cell death (PCD) [20]. PCD is a type of cell death that proceeds through controlled pathways under stress [21]. In eukaryotic systems, including yeast, multiple PCD-like phenotypes, such as apoptosis-like death, have been discussed [22]. In 2012, Dixon *et al*. introduced ferroptosis as a distinct form of regulated cell death [23]. In mammalian cells, ferroptosis is characterized by iron-dependent lipid peroxidation, in which ROS and redox-active iron promote the peroxidation of membrane phospholipids [24]. Particularly those containing polyunsaturated fatty acids (PUFAs), resulting in the accumulation of lipid hydroperoxides [25]. As lipid hydroperoxides accumulate, membrane integrity and membrane-associated functions decline [26]. This damage can become irreversible and result in cell death [27]. Thus, ferroptosis reflects a convergence of ROS, iron, and lipid peroxidation [24]. Similarly, ferroptosis-like responses have also been reported in microorganisms, where increased intracellular iron accumulation and lipid peroxidation are observed [28]. Recent studies have reported a link between hesperetin and ferroptosis in mammalian cell models [29]. However, the mechanisms underlying the effects of hesperetin in eukaryotic microorganisms remain poorly understood. Among these organisms, *S. cerevisiae* is one of the best-characterized model systems. It is a non-pathogenic, genetically tractable eukaryotic model with well-characterized redox and iron homeostasis networks [30, 31]. Therefore, in this study, *S. cerevisiae* was used as a model organism to determine whether hesperetin can trigger a ferroptosis-like response in this yeast.

## Material and Methods

### Material Preparation

In this experiment, hesperetin (Sigma Aldrich, USA), a natural flavonoid with antimicrobial properties, was used to evaluate its antifungal effect on *Saccharomyces cerevisiae* (KCTC 7296) obtained from the Korean Collection for Type Cultures (KCTC, Republic of Korea). Norfloxacin (USA), a fluoroquinolone antibiotic, served as a positive control although primarily antibacterial, it has shown antifungal activity against *S. cerevisiae*. Ferrostatin-1 (Sigma Aldrich) and Erastin (Sigma Aldrich) were used to investigate the role of Ferroptosis-like death.

### Detection of Intracellular ROS Levels

Dihydroethidium (DHE, Sigma Aldrich) was used to detect H_2_O_2_ or O_2_^-^ generation and 3′-(p-hydroxyphenyl) (HPF, Molecular Probes, USA) was used to detect OH∙ generation. Cells were cultured in YPD medium at 28°C overnight and then resuspended in phosphate-buffered saline (PBS). Cells (1 × 10^5^ cells/ml) were treated with hesperetin, norfloxacin and pretreated with ferrostatin-1. Cells were incubated at 28°C for 4 h and then centrifuged at 8,000 g for 5 min. After DHE and HPF were added to each cell and incubated at 28°C for 30 min, the fluorescence of each regent was analyzed using a FACSverse flow cytometer (Becton Dickinson, USA).

### Measurement of Glutathione (GSH) Depletion

To measure the quantitative glutathione (GSH) concentration, Cells were treated with hesperetin, norfloxacin and erastin, and some of them were pretreated with ferrostatin -1. for 4 h and 5% 5-sulfosalicylic acid for protein precipitation was added to the pellets. Then, beads for cell lysis were put on the pellets and incubated for 5 min at 4°C. The samples were centrifuged and the supernatant was used for measurement. Then, 2-vinylpyridine were added at room temperature for 1 h and centrifuged. The absorbance of the supernatant was measured with a microtiter ELISA reader at 415 nm. Each protein concentration was assessed through the Bradford method and depletion of glutathione was observed after normalizing to total protein level.

### Measurement of Iron Accumulation

Overloading of iron was measured accordingly using an iron assay kit following the manufacturer’s instructions (Sigma). Cells were treated with hesperetin, erastin, and some of them were pretreated with ferrostatin-1. Samples were incubated for 4 h at 28°C, centrifuged at 8,000 g for 5 min, and the supernatants were removed. The cell pellet was resuspended in assay buffer for five times the volume of the pellet. Then, 0.4 g beads were added to the samples, and vortexed for 1 min and 30 sec and frozen for 1 min, which was repeated four times. Supernatants were collected, and 50 μl of each supernatant was suspended in a 96-well plate, and the same volume of iron assay buffer was added. Then, 5 μl of iron reducer was added and incubated for 30 min, followed by treatment with 100 μl of iron probe and incubated for 1 h. The absorbance was analyzed at 600 nm, and the total iron levels were calculated according to standard curves.

### Observation of Lipid Peroxidation

The levels of malondialdehyde (MDA), which indicates the degree of oxidative stress, were measured using the thiobarbituric acid reactive substances assay to assess lipid peroxidation. *S. cerevisiae* was cultured overnight in YPD medium at 28°C and resuspended in PBS. Cells were treated with hesperetin, erastin, and some of them were pretreated with ferrostatin-1. Samples were incubated in a water bath for 4 h. Cells were sonicated for 1 min in a lysis buffer (10 mM Tris–HCl, pH 8.0, 1% SDS, 1 mM EDTA, 2% Triton X-100, and 10 mM NaCl) and centrifuged at 8,000 g for the collection of the supernatant. Next, 5% thiobarbituric acid solution was added, and the solution was heated at 95°C. After cooling at room temperature for 5 min, the levels of MDA were measured at 532 nm using a spectrophotometer (DU530, Beckman, USA).

### Evaluation of Membrane Integrity

To assess membrane integrity using Bis-(1,3-Dibutylbarbituric acid) Trimethine Oxonol (DiBAC_4_(3), Molecular Probes) and propidium iodide (PI). Cells were incubated with hesperetin, erastin, or ferrostatin-1 pretreatment with hesperetin at 28°C for 4 h. Then, the samples were centrifuged at 8,000 g and the pellets were resuspended with PBS and stained with DiBAC_4_(3) and PI. The fluorescence intensity of the samples was observed using an FACSVerse flow cytometer.

### Detection of Metacaspase Activity

Metacaspase activation was detected using the CaspACE FITC-VAD-FMK In Situ Marker (Promega, USA). Cells were incubated with hesperetin, norfloxacin and erastin at 28°C. After 4 h of incubation, the cells were washed twice with PBS, incubated with CaspACE FITC-VAD-FMK (50 μM) for 30 min, centrifuged at 8,000 g for 5 min, and resuspended in PBS. Fluorescence was measured using a flow cytometer.

### Estimation of DNA Fragmentation

DNA fragmentation was analyzed using the terminal deoxynucleotidyl transferase dUTP nick end labeling (TUNEL) assay with an In Situ Cell Death Detection Kit (Roche Applied Science, Switzerland). For this assay, cells were treated with hesperetin, norfloxacin and erastin and incubated for 2 h at 28°C. The cells were then washed with PBS and fixed with 2% paraformaldehyde on ice for 1 h. Subsequently, the fixed cells were permeabilized using a solution containing 0.1% Triton X-100 and 0.1% sodium citrate on ice for 2 min, followed by incubation with the TUNEL reaction mixture for 1 h at 37°C. Fluorescence intensity for each sample was measured using a flow cytometer.

### Examination of Ferroptosis-Like Response

N -(4-diphenylphosphinophenyl)- N -(3,6,9,12- tetraoxatridecyl) perylene-3,4,9,10 tetracarboxydiimide (Liperfluo, Dojindo, Japan) probe can be used to detect ferroptosis-like death in fungi by reacting with lipid peroxidase, which promotes ferroptosis in the cell). Cells were incubated with hesperetin, norfloxacin, erastin and ferrostatin-1 pretreatment with hesperetin. Cells were incubated for 4 h at 28°C, washed twice with PBS, and the fluorescence intensity was measured using a flow cytometer.

### Statistical Analysis

All the experiments were performed in triplicate, and the values were expressed as the means ± standard deviation. After confirming the normality of distribution using Shapiro–Wilk tests, statistical comparisons between various groups were carried out using analysis of variance followed by Tukey's post-hoc test for three-group comparisons using SPSS software (version 25, SPSS/IBM, USA). Intergroup differences were considered statistically significant at *p* <0.05.

## Results

### Detection of Intracellular ROS

**Fig. 1:**
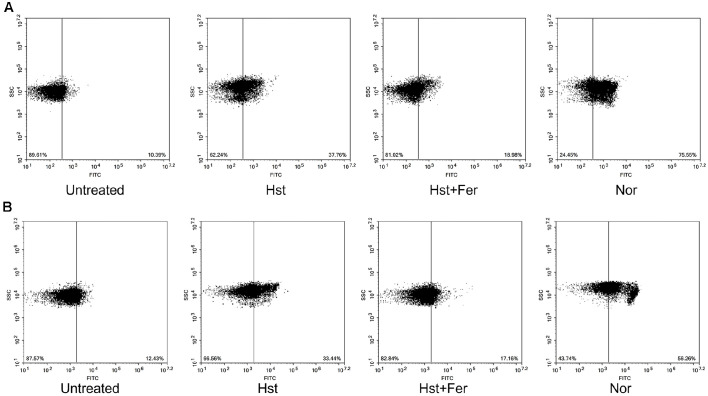
Estimation of ROS generation. (**A**) DHE (**B**) HPF Sample was treated with hesperetin (2.5 mM), Norfloxacin (0.15 μg/ml), ferrostatin-1 (1 μM), Erastin (10 μM) Experiments were conducted in triplicate independently.

Intracellular ROS accumulation is commonly used as an indicator of cellular stress. To investigate the relationship between hesperetin treatment and ROS generation, ROS levels were measured using the DHE and HPF assays. In the DHE assay, DHE is oxidized by superoxide to form ethidium. Oxidized ethidium then binds to DNA and emits red fluorescence (Fig. 1A). Untreated cells showed 10.39% fluorescence positive cells. In contrast, cells treated with hesperetin or norfloxacin showed increased signals of 37.76% and 75.55%, respectively. Ferrostatin-1 treatment reduced the signal to 18.98%, indicating suppression of ROS generation. In the HPF assay, fluorescence is produced in the presence of hydroxyl radicals (Fig. 1B). Untreated cells showed 12.43% fluorescence positive cells. Hesperetin and norfloxacin treated cells exhibited higher signals of 33.44% and 56.26%, respectively. By contrast, ferrostatin-1 reduced the signal to 17.16%, suggesting inhibition of hydroxyl radical production. These results indicate that hesperetin increases intracellular ROS levels.

### Depletion of Intracellular GSH

**Fig. 2:**
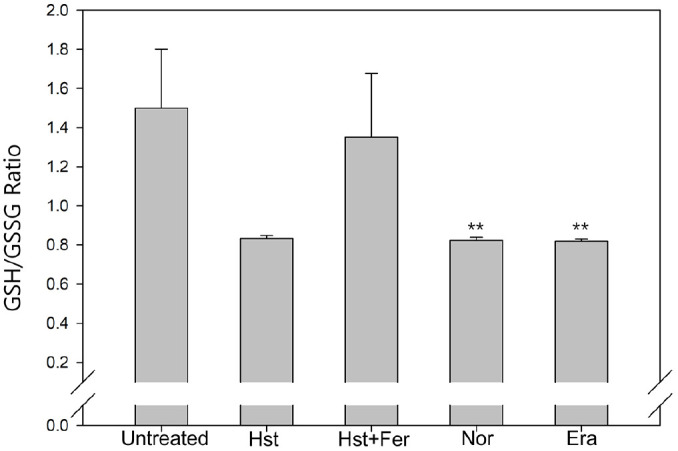
Assesment of glutathione depletion GSH/GSSG ratio. Sample was treated with hesperetin (2.5 mM), Norfloxacin (0.15 μg/ml), ferrostatin-1 (1 μM), Erastin (10 μM) Experiments were conducted in triplicate independently the average, standard deviation, and *p* values from three experiments (*p < 0.1; **p < 0.05; ***p < 0.01 vs. untreated sample). Statistical analysis was performed utilizing Tukey's test.

Intracellular GSH is a tripeptide that plays central roles in detoxifying ROS and peroxides and maintaining cellular redox balance. When ROS accumulate, GSH consumption increases and a larger fraction of glutathione is converted to the oxidized form, GSSG. This shifts the intracellular redox state toward a more oxidizing condition. Consequently, the GSH/GSSG ratio decreases. This ratio is widely used as an indicator of the extent of oxidative stress in cells (Fig. 2). Cells treated with hesperetin, norfloxacin, and erastin exhibited lower GSH/GSSG ratios (0.83, 0.82, and 0.82, respectively) than untreated cells (1.5). However, ferrostatin-1 treatment increased this ratio to 1.35. These results indicate that ROS accumulation induced by hesperetin is associated with the depletion of GSH and disruption of GSH-dependent redox homeostasis.

### Overloading of Intracellular Iron

**Fig. 3:**
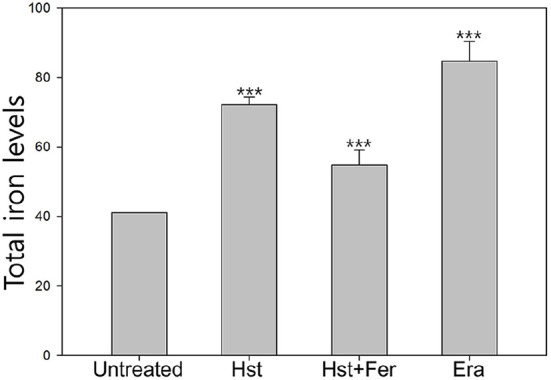
Detection of iron accumulation Sample was treated with hesperetin (2.5 mM), ferrostatin-1 (1 μM), Erastin (10 μM) Experiments Experiments were conducted in triplicate independently the average, standard deviation, and *p* values from three experiments (*p < 0.1; **p < 0.05; ***p < 0.01 vs. untreated sample). Statistical analysis was performed utilizing Tukey's test.

Iron is essential for cellular physiology, serving as a key cofactor in electron transportation, energy production, and numerous enzymatic reactions, it can also act as a risk factor that amplifies oxidative stress (Fig. 3). Compared with untreated cells with an intracellular iron level of 41, cells treated with hesperetin or erastin showed increased iron levels of 72.2 and 89.6, respectively, indicating iron accumulation under these conditions. In contrast, ferrostatin-1 treatment reduced the iron level to 54.7, suggesting suppression of iron accumulation. This result supports the interpretation that hesperetin induced oxidative stress is associated with increased intracellular iron accumulation.

### Estimation of MDA Levels

**Fig. 4:**
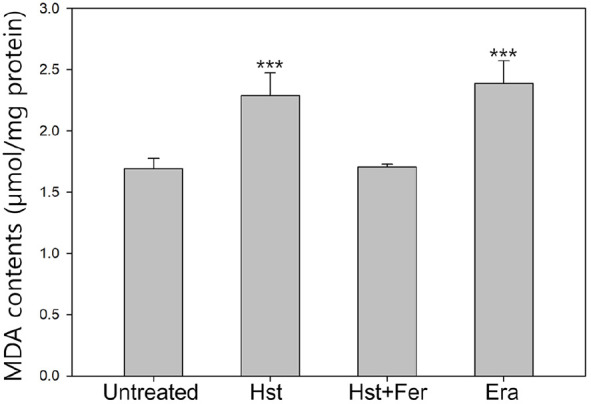
Observation of MDA level Sample was treated with hesperetin (2.5 mM), ferrostatin-1 (1 μM), Erastin (10 μM) Experiments Experiments were conducted in triplicate independently the average, standard deviation, and *p* values from three experiments (*p < 0.1; **p < 0.05; ***p < 0.01 vs. untreated sample). Statistical analysis was performed utilizing Tukey's test.

Lipid peroxidation is a chain reaction in which membrane phospholipids are oxidized by ROS, resulting in the formation of lipid hydroperoxides. As these lipid hydroperoxides decompose, various aldehyde byproducts are generated. Of these, malondialdehyde (MDA) is a representative end product. Therefore, the MDA level is commonly used as an indirect indicator of lipid peroxidation (Fig. 4). Compared with untreated cells with an MDA level of 1.69, cells treated with hesperetin or erastin showed increased levels of 2.3 and 2.4, respectively, suggesting enhanced lipid peroxidation. In contrast, treatment with ferrostatin-1, a lipid ROS scavenger, reduced the MDA level to 1.7, indicating suppression of lipid peroxidation. This result indicates that hesperetin induces lipid peroxidation.

### Detection of Membrane Damage

**Fig. 5:**
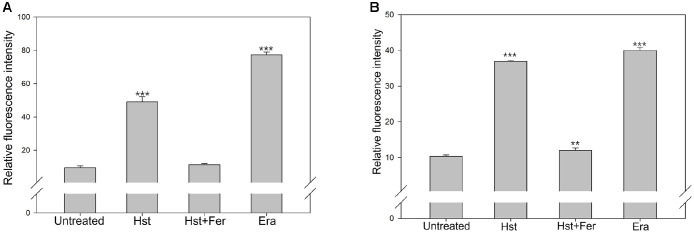
Analysis of membrane damage (A) DiBAC_4_(3) (B) PI Sample was treated with hesperetin (2.5 mM), ferrostatin-1 (1 μM), Erastin (10 μM) Experiments Experiments were conducted in triplicate independently the average, standard deviation, and *p* values from three experiments (*p < 0.1; **p < 0.05; ***p < 0.01 vs. untreated sample). Statistical analysis was performed utilizing Tukey's test.

Membrane integrity and membrane potential were assessed using propidium iodide (PI) and DiBAC_4_(3) staining. In the DiBAC_4_(3) assay, membrane depolarization promotes dye entry and intracellular accumulation, resulting in increased fluorescence (Fig. 5A). Compared with untreated cells at 9.84%, cells treated with hesperetin or erastin showed markedly higher DiBAC_4_(3) positive populations of 49.56% and 78.2% respectively, indicating substantial membrane depolarization. In contrast, ferrostatin-1 treatment reduced the DiBAC_4_(3) positive population to 10.94%, suggesting suppression of depolarization. PI staining was used to evaluate membrane integrity because PI enters cells only when the plasma membrane is compromised and then binds DNA to emit fluorescence (Fig. 5B). Compared with untreated cells at 10.12%, PI positive populations increased to 36.71% and 40.52% in hesperetin and erastin treated cells, respectively, indicating loss of membrane integrity. Ferrostatin-1 treatment reduced the PI positive population to 11.78%, consistent with inhibition of membrane damage. These results indicate that hesperetin induces membrane dysfunction and damage, and that this phenotype is attenuated by ferrostatin-1.

### Absence of Apoptotic Hallmarks

**Fig. 6:**
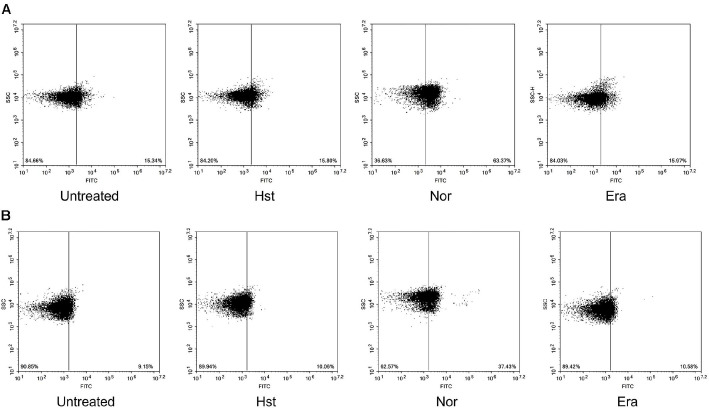
Measurement of apoptosis-like death hallmarks. (**A**) CaspACE FITC-VAD-FMK (**B**) TUNEL Sample was treated with hesperetin (2.5 mM), Norfloxacin (0.15 μg/ml), Erastin (10 μM) Experiments were conducted in triplicate independently.

Apoptosis is typically associated with hallmark features such as caspase activation and DNA fragmentation. In contrast, ferroptosis is primarily characterized by iron-dependent lipid peroxidation rather than caspase activation or DNA fragmentation. Compared with untreated cells at 15.34%, norfloxacin markedly increased the caspase-activated population to 64.06%, whereas hesperetin and erastin treated cells showed values of 15.8% and 15.97%, indicating no appreciable caspase activation under these conditions (Fig. 6A). Similarly, in the TUNEL assay, norfloxacin increased TUNEL positive cells to 37.43% compared with 9.51% in untreated cells, consistent with pronounced DNA fragmentation. In contrast, hesperetin and erastin treated cells showed only 10.61% and 10.58% TUNEL positivity, indicating little to no DNA fragmentation (Fig. 6B). These results indicate that hesperetin does not induce apoptosis hallmarks, including caspase activation and DNA fragmentation.

### Determination of Ferroptosis-Like Response

**Fig. 7:**
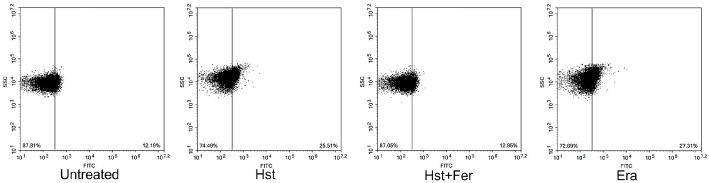
Determination of Ferroptosis-lke response by using Liperfluo. Sample was treated with hesperetin (2.5 mM), ferrostatin-1 (1 μM), Erastin (10 μM) Experiments Experiments were conducted in triplicate independently.

Lipid peroxidation leads to the formation of lipid hydroperoxides. Liperfluo staining is a lipid peroxide–responsive dye, and an increase in Liperfluo fluorescence indicates elevated lipid hydroperoxide accumulation, which represents a key biochemical feature of ferroptosis (Fig. 7). Compared with untreated cells at 12.19% and 11.25%, cells treated with hesperetin or erastin showed increased Liperfluo positive populations of 25.51% and 27.31%, respectively, indicating enhanced lipid hydroperoxide accumulation. In contrast, ferrostatin-1 treatment reduced the Liperfluo positive population to 12.95%, consistent with suppression of lipid peroxidation. This result indicates that hesperetin promotes lipid hydroperoxide accumulation and supports a ferroptosis-like response in *S. cerevisiae*.

## Discussion

Flavonoids are widely present in foods and show diverse biological activities [32]. Among them, hesperetin has been reported to provide physiological benefits and to exhibit antimicrobial activity [6]. It can also modulate redox balance and, in some settings, increase intracellular reactive oxygen species [33]. ROS are produced when oxygen is incompletely reduced during metabolism [34]. Electron leakage from the mitochondrial electron transport chain is a major source, generating reactive oxidants and radicals [35]. When ROS levels overwhelm antioxidant defenses, inducing oxidative stress and cellular damage [36]. This condition promotes protein oxidation and membrane damage and can push cells toward irreversible dysfunction and death [19, 37]. In this study, hesperetin increased intracellular ROS levels in *S. cerevisiae*, indicating the generation of oxidative stress. ROS accumulation can overwhelm and ultimately disrupt cellular antioxidant defenses [38].

Cells control ROS through multiple antioxidant systems, including GSH [39]. Reduced GSH neutralizes reactive species and peroxides, converting to GSSG [40]. Glutathione reductase then regenerates GSH using NADPH, maintaining redox buffering capacity and preserving a reducing intracellular environment [41]. Under excessive oxidative pressure, GSH can be rapidly depleted, and redox enzymes can become less effective, further weakening ROS clearance [18, 42]. Moreover, a decrease in the GSH/GSSG ratio reflects a shift in the cellular redox poise toward a more oxidizing state and often coincides with impaired detoxification of peroxides and increased susceptibility to downstream oxidative damage [43, 44]. In this study, hesperetin decreased the GSH/GSSG ratio, suggesting the disruption of GSH-dependent redox homeostasis and weakening of antioxidant defense.

ROS-mediated damage is intensified by iron-dependent redox reactions [45]. Therefore, tight regulation of iron homeostasis is essential for cellular survival [46]. In mammalian cells, iron homeostasis is regulated by coordinated control of iron uptake, storage, and export through the IRP/IRE system [47]. In *S. cerevisiae*, Aft1/Aft2 regulates iron acquisition pathways, while Yap5/Ccc1 promotes vacuolar sequestration under high-iron conditions [48]. Although these frameworks differ, both systems ultimately limit redox-active iron availability. In the presence of H_2_O_2_ and redox-active Fe^2+^, Fenton chemistry generates highly reactive •OH [49]. The generated •OH can initiate oxidative chain reactions and markedly increase cellular injury [50]. Moreover, •OH can initiate lipid peroxidation by abstracting hydrogen atoms from membrane phospholipids, especially those containing PUFAs [51]. Once initiated, lipid peroxidation propagates as a chain reaction that yields lipid peroxyl radicals and lipid hydroperoxides and spreads damage across membranes [52]. Accumulated lipid hydroperoxides are chemically unstable and can decompose into reactive electrophilic aldehydes, including MDA [53]. MDA is widely used as an indirect marker of oxidative lipid damage [54]. This study demonstrates that hesperetin expanded the pool of Fe^2+^ capable of reacting with ROS and increased MDA accumulation, suggesting enhanced lipid peroxidation. When lipid peroxidation exceeds a critical threshold, the membrane barrier collapses [55]. This leads to loss of membrane potential and increased membrane fluidity, resulting in compromised membrane integrity [56]. Beyond this point, recovery is often difficult, and the process readily progresses to the execution phase of cell death [57].

When cell death is induced, it is accompanied by various cellular changes, and the characteristic hallmarks vary depending on the mode of death [58]. Apoptosis is characterized by caspase activation and DNA fragmentation, whereas ferroptosis is characterized by iron-dependent lipid peroxidation rather than apoptosis-associated features [22, 59]. This study demonstrates that caspase activation and DNA fragmentation were not detected after hesperetin treatment, arguing against a dominant apoptosis-like mechanism. Instead, the combination of ROS accumulation, increased Fe^2+^ availability, lipid peroxidation, and suppression by ferrostatin-1 supports a ferroptosis-like response in this study. Ferrostatin-1 is a radical-trapping antioxidant that inhibits lipid peroxidation chain reactions, and its protective effect suggests that lipid oxidation is functionally important rather than a passive byproduct [60]. Overall, the findings support a model in which hesperetin disrupts glutathione-based redox buffering, promotes iron-dependent radical chemistry, drives lipid peroxidation, and triggers membrane dysfunction that culminates in ferroptosis-like response in *S. cerevisiae*.

In summary, hesperetin promotes intracellular ROS accumulation. Excess ROS is accompanied by glutathione depletion and an increase in reactive iron. The expanded Fe^2+^ pool can drive Fenton chemistry to generate highly reactive hydroxyl radicals. These radicals can attack membrane phospholipids and initiate lipid peroxidation. If lipid peroxidation persists, membrane damage accumulates and can ultimately lead to cell death. Notably, apoptotic hallmarks such as caspase activation and DNA fragmentation were not observed in this study. In contrast, lipid hydroperoxide accumulation, a key feature of ferroptosis, was induced and was suppressed by the ferroptosis inhibitor ferrostatin-1. Together, these findings indicate that hesperetin triggers a ferroptosis-like response in *S. cerevisiae*.
